# Comparison of Accuracy of Conventional Periapical Radiography and Direct Digital Subtractions Radiography with or without Image Enhancement in the Diagnosis of Density Changes

**DOI:** 10.5681/joddd.2012.012

**Published:** 2012-06-06

**Authors:** Tahmineh Razi, Arezu Mohammadi, Morteza Ghojazadeh

**Affiliations:** ^1^Assistant Professor, Department of Oral and Maxillofacial Radiology, Faculty of Dentistry, Tabriz University of Medical Sciences, Tabriz, Iran; ^2^DDS, Private Practice, Tabriz, Iran; ^3^Assistant Professor, Department of Physiology, Faculty of Medicine, Tabriz University of Medical Sciences, Tabriz, Iran

**Keywords:** Density, direct digital subtraction radiography, image enhancement, periapical radiography

## Abstract

**Background and aims:**

In periapical radiographic technique, the changes will be visible only after considerable deposi-tion or resorption while digital subtraction technique visualizes slight density changes. This study was aimed to compare visualization of density changes in conventional periapical radiographs and digital subtraction technique with or without image enhancement.

**Materials and methods:**

Three dry human mandibles with unspecified age and gender were selected. Conventional periapical and direct digital radiographs were taken from the anterior, and right and left posterior regions by step-wise placement of aluminum plates until the image of the plate was clearly visible. The radiographs taken with the direct digital technique were subtracted from the first radiograph using Photoshop software. Three observers evaluated the radiographs and the digital subtraction images with or without image enhancement. The density was recorded in each radiograph in which the image of the aluminum plate was completely visible.

**Results:**

In all mandibles, the differences in diagnosis of densitychanges between the conventional periapical radiographic technique and the direct digital subtraction radiographic technique with or without image enhancement were statistically significant irrespective of the region under study (p<0.001). There were no significant differences in the diagnosis of density changes in all the three mandibles in the left and right posterior regions between the two radiographic techniques. However, the differences in the anterior region were statistically significant (p<0.001).

**Conclusion:**

Direct digital subtraction radiographic technique with or without image enhancement is a more efficacious technique in exhibiting minor density changes compared to conventional periapical radiographic technique.

## Introduction

Despite high resolution of periapical radiographs, time is needed to visualize changes in density in these radiographs and the great amount of mineral loss might influence treatment prognosis. For example, a period of 6–8 months is necessary to visualize changes in mild periodontitis.^1 , 2^ In digital subtraction radiographic technique, changes up to even 5% are visible and a much shorter time is needed to visualize changes, which is an advantage of the technique.^3^ In addition, the technique enables the dental practitioner to manipulate the contrast and brightness in over-exposed and under-exposed radiographs to diagnose osseous and carious lesions.^4^

Digital radiographic techniques have been used in dental practice for more than 25 years but they have not completely replaced conventional radiographic technique.^4^

Hildebolt evaluated alveolar bone changes by the use of bitewing (BW) radiographic technique, digital BW radiographic technique and digital enhancement BW technique and reported that all the three radiographic techniques yield similar results in the diagnosis of vertical lesions due to low sensitivity, with the digital enhancement BW technique having the highest validity in the diagnosis of bone loss.^5^ In the study mentioned, digitized radiographs were evaluated. Miguens did not report any differences in the diagnosis of periapical lesions between digital panoramic radiographs and digitized panoramic radiographs with the use of digital subtraction technique on dry skulls.^6^ The results might be attributed to the use of panoramic images with low resolution compared to periapical images; in addition, the digital panoramic images were indirect, which might have exerted an influence on the results. In the present study, conventional and digital subtraction radiographic techniques were compared in an attempt to evaluate density changes by step-wise addition of aluminum plates.

## Materials and Methods

**Figure 1:**
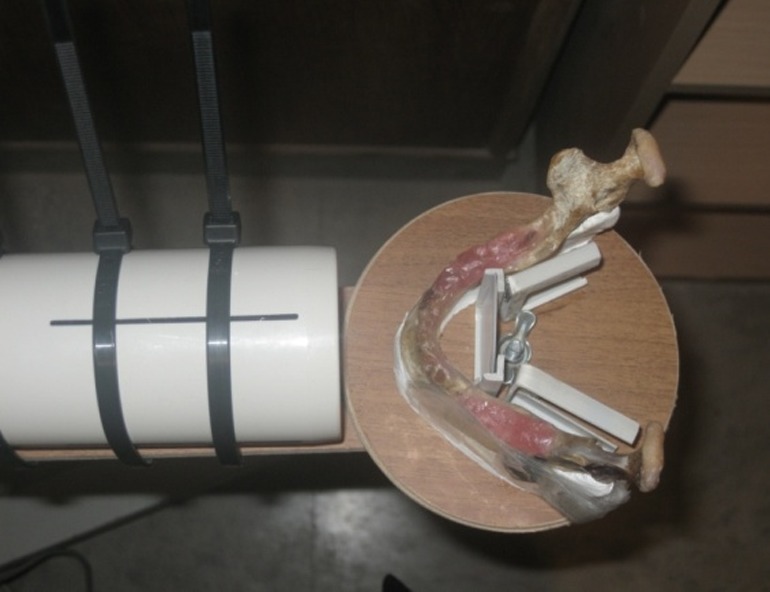
The mandible and the x-ray tube mounted on the holder seen from above.

**Figure 2:**
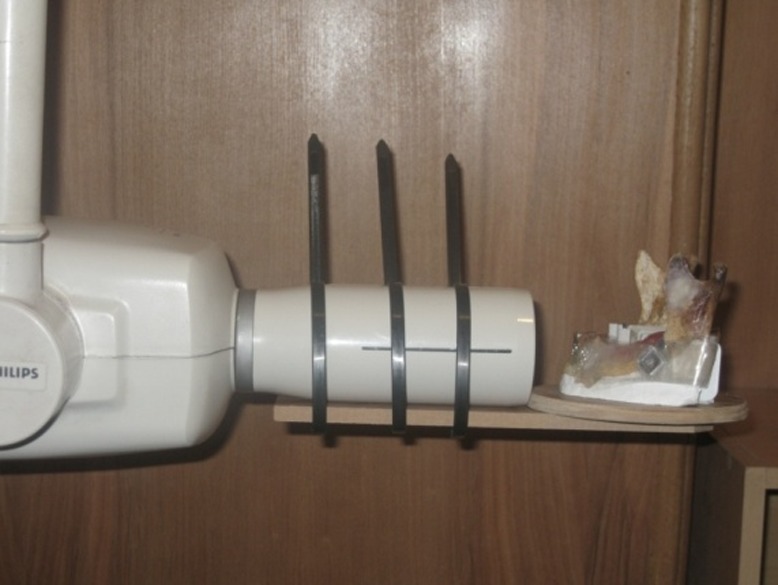
The mandible and the x-ray tube mounted on the holder (lateral view).

In this descriptive–analytical study, three dry edentulous human mandibles with unspecified age and gender were selected. Panoramic radiographs of the mandibles were provided and no pathologic lesions were detected. A hand-made gypsum plaster holder was used to fixate the mandibles and align the tube head parallel to the regions to be radiographed. PVC was used to fabricate three film holders to hold the films parallel to the tube and the mandibles (Figures 1 and 2).

The radiographs were taken with an intraoral radiographic machine (ORALIX 65S, Philips, Italy) in the Radiology Department, Faculty of Dentistry, Tabriz University of Medical Sciences. Square (10 mm × 10 mm) and triangular (13 mm × 10 mm ×10 mm) aluminum plates with a thickness of 33±2 μ were provided. E-speed films (Kodak, France) were used to take radiographs from the right and left molar and anterior regions of the mandibles using film holders and the mandible holding device in paralleling technique under the same radiographic condition (mA=7.5, kVp=65, time=0.8 seconds). At first a periapical radiograph and a direct digital radiograph were taken in the anterior region without placing aluminum plates; Kodak RVG (5100, UK) was used for the direct digital technique.

Then an aluminum plate with a thickness of 33±2 μ and a known geometric form was placed in the region and the same radiographs were taken. Consecutive periapical and direct digital radiographs were taken by step-wise addition of aluminum plates to the point at which the complete form of the aluminum plate was clearly visible in the periapical radiograph. The steps were repeated for the right and left posterior regions. All the films were processed in one session using an automatic film processor (Velopex Extra-X, UK). Then the radiographs were numbered. CS3 Photoshop software was used to subtract the radiograph taken with the direct digital technique from the first radiograph taken from the same region.

Three pre-instructed observers visualized the periapical and subtracted radiographs with and without image enhancement. Image enhancement procedures were carried out by changing the contrast of the films by brightness/contract tools of Photoshop software on the subtracted image, with no changes in the film density. When the full shape of the aluminum plate was visualized on each film, the density was determined. At this stage, the thickness of the aluminum plate was separately recorded for periapical and the digitally subtracted radiographs. After data collection, the results of density at the specified aluminum plate thicknesses were compared between periapical radiographs and direct digital subtraction images with and without image enhancement.

The collected data were analyzed using descriptive statistical methods (means and standard deviations), one- way ANOVA and a post hoc Turkey test. Statistical significance was defined at p<0.05 level.

Evaluation of the observers was carried out by Kappa correlation coefficient, which was at a very high level.

## Results

One-way ANOVA revealed statistically significant differences in the visualization of density changes regarding the number of aluminum plates in the three mandibles between the three radiographic techniques of periapical and direct digital subtraction with or without image enhancement (p<0.001). The results of Tukey test reveled statistically significant differences in the visualization of density changes between the three mandibles in the two-by-two comparisons (p<0.001) (Tables 1 and 2).

**Table 1:** Descriptive statistics of the number of aluminum plates in relation to the visualization of density changes in the three mandibles under study

Studied mandible	Mean ± SEM	Minimum number	Maximum number	P
Mandible 1				
CONV DSR EDSR	41.33±5.27 21.78±4.33 20.11±4.48	16 7 5	61 38 36	<0.001
Mandible 2				
CONV DSR EDSR	20.56±1.62 5.78±0.45 4.11±0.20	13 5 3	26 8 5	<0.001
Mandible 3				
CONV DSR EDSR	20.67±2.05 7.44±0.37 4.44±0.50	11 6 2	29 9 6	<0.001

CONV: Conventional radiography; DSR: Digital subtraction radiography without enhancement; EDSR: Digital subtraction radiography with enhancement.

**Table 2:** Descriptive statistics of the number of aluminum plates and the visualization of density changes in relation to the observers irrespective of the area and the mandible under study

Observer	Mean ± SEM	Minimum number	Maximum number	p
1				
CONV DSR EDSR	26.22±5.06 11.56±3.57 9.33±3.78	13 5 2	59 37 36	<0.001
2				
CONV DSR EDSR	29.22±4.40 12.00±3.62 9.89±3.63	14 5 3	59 38 36	<0.001
3				
CONV DSR EDSR	27.11±4.98 11.44±3.54 9.44±3.70	11 5 2	61 37 36	<0.001

CONV: Conventional radiography; DSR: Digital subtraction radiography without enhancement; EDSR: Digital subtraction radiography with enhancement.

The results revealed statistically significant differences in the visualization of density changes in relation to the number of aluminum plates between the three observers (p<0.001).

Regarding observer 1, the results showed significant differences in the visualization of density changes in the two-by-two comparison of the radiographic techniques (p<0.001).

Reading observers 2 and 3, the results showed significant differences in the visualization of density changes in the two-by-two comparison of the radiographic techniques (p<0.001), except for the comparison between the direct digital subtraction techniques with and without image enhancement. Table 3 presents the descriptive statistics in relation to the number of aluminum plates.

**Table 3:** Descriptive statistics in relation to the number of aluminum plates and visualization of density changes in the regions under study

Region	Mean ± SEM	Minimum number	Maximum number	p
Anterior				
CONV DSR EDSR	24.56±1.26 7.56±0.24 4.33±0.47	16 7 2	29 9 6	<0.001
Posterior right				
CONV DSR EDSR	31.22±7.21 10.67±2.51 9.44±2.48	11 5 3	61 21 20	<0.001
Posterior left				
CONV DSR EDSR	26.78±3.73 16.78±5.16 14.89±5.28	13 5 4	43 38 36	<0.001

CONV: Conventional radiography; DSR: Digital subtraction radiography without enhancement; EDSR: Digital subtraction radiography with enhancement.

The results showed statistically significant differences in the visualization of density changes between the anterior and left and right posterior regions (p<0.001). In addition, there were significant differences in the two-by-two comparison of the radiographic techniques (p<0.001), except for the mandibular right and left posterior regions, which did not reveal statistically significant differences between the two digital subtraction radiographic techniques with and without image enhancement.

## Discussion

The results of the present study showed that the direct digital subtraction radiographic technique with image enhancement helps to visualize minor density changes, which hold for all the regions except for the right and left mandibular posterior regions, in which the differences between the two direct digital subtraction techniques with and without image enhancement were not statistically significant. This finding might be attributed to the high density of the posterior region of the mandible, which prevents the visualization of minor density changes by this technique. It seems that the techniques used in the present study do not reveal minor density changes in areas with high osseous density. It should be pointed out that in the present study the radiographic techniques used to evaluate density changes relied on increasing the density step by step; i.e. adding aluminum plates simulated the deposition of minerals in osseous tissues. In the present study, the mean of aluminum plate thicknesses were higher in mandible 1 than those in mandibles 2 and 3. The mean of aluminum plate thicknesses in a study carried out by Christagau was less than that in the present study,^7^ which is attributed to the bigger size of mandible 1 and its higher bone density in the present study, making the aluminum plates visible at higher thicknesses.

Masood et al^8^ achieved lower accuracy on conventional panoramic radiographs compared to digital subtraction technique with and without image enhancement in the evaluation of osteophytic lesions of mandibular condyle. They used bone chips with 0.5-1 mm in thicknesses. Miguens^9^ did not report any significant differences between digital and digitized panoramic radiographs using subtraction techniques in the evaluation of periapical lesions, which is attributed to the low resolution of panoramic radiographs compared to the high resolution of periapical radiographs. Therefore, subtraction technique is more efficacious with periapical radiographs. Based on the results of the present study, it was concluded that the diagnostic value of direct digital subtraction technique with and without image enhancement is more than that of conventional radiographic technique in evaluation of density changes. The use of brightness/contrast tool of Photoshop software results in the visualization of density changes with less layers of aluminum plates.
